# Molecular Mechanisms of Cardiac Adaptation After Device Deployment

**DOI:** 10.3390/jcdd12080291

**Published:** 2025-07-30

**Authors:** Letizia Rosa Romano, Paola Plutino, Giovanni Lopes, Rossella Quarta, Pierangelo Calvelli, Ciro Indolfi, Alberto Polimeni, Antonio Curcio

**Affiliations:** 1 Division of Cardiology, Annunziata Hospital, 87100 Cosenza, Italy; 2 Department of Pharmacy, Health and Nutritional Sciences, University of Calabria, 87036 Rende, Italy

**Keywords:** cardiac device, myocardial remodeling, heart failure, ventricular arrhythmias, ischemic cardiomyopathy

## Abstract

Cardiac devices have transformed the management of heart failure, ventricular arrhythmias, ischemic cardiomyopathy, and valvular heart disease. Technologies such as cardiac resynchronization therapy (CRT), conduction system pacing, left ventricular assist devices (LVADs), and implantable cardioverter-defibrillators have contributed to abated global cardiovascular risk through action onto pathophysiological processes such as mechanical unloading, electrical resynchronization, or hemodynamic optimization, respectively. While their clinical benefits are well established, their long-term molecular and structural effects on the myocardium remain under investigation. Cardiac devices dynamically interact with myocardial and vascular biology, inducing molecular and extracellular matrix adaptations that vary by pathology. CRT enhances calcium cycling and reduces fibrosis, but chronic pacing may lead to pacing-induced cardiomyopathy. LVADs and Impella relieve ventricular workload yet alter sarcomeric integrity and mitochondrial function. Transcatheter valve therapies influence ventricular remodeling, conduction, and coronary flow. Understanding these remodeling processes is crucial for optimizing patient selection, device programming, and therapeutic strategies. This narrative review integrates the current knowledge on the molecular and structural effects of cardiac devices, highlighting their impact across different disease settings.

## 1. Introduction

The implantation of cardiac devices has revolutionized the management of heart failure (HF), ventricular arrhythmias (VAs), valvular heart disease (VHD), and ischemic cardiomyopathy, significantly improving survival and quality of life in affected patients [1]. While the mechanical and electrical functions of these devices are well characterized, their long-term biological effects on the myocardium remain an evolving field of study [2]. Chronic interaction between implanted devices and the cardiac environment triggers a complex network of molecular, cellular, and structural adaptations that could influence disease progression and therapeutic outcomes [3]. These changes vary according to the underlying pathology, shaping both adaptive and maladaptive myocardial responses [4,5].

The interplay between cardiac devices and myocardial biology is a dynamic process influenced by time-dependent changes in gene expression, protein regulation, and extracellular matrix (ECM) remodeling [6]. Cardiac resynchronization therapy (CRT), left ventricular assist devices (LVADs), and short-term circulatory support devices impose different biomechanical and electrophysiological stresses on the heart, triggering diverse molecular cascades that can either facilitate recovery or exacerbate pathological adaptations. Mechanical unloading with LVADs and short-term support devices like Impella reduces ventricular workload and improves hemodynamics but may lead to myocardial atrophy by downregulating contractile proteins and disrupting sarcomere integrity. This raises questions about the reversibility of these changes and the potential role of pharmacological or genetic interventions in enhancing myocardial recovery post-device implantation [7,8]. While CRT has been shown to promote reverse remodeling through improved calcium handling and reduced fibrosis [9], chronic pacing can paradoxically lead to pacing-induced cardiomyopathy (PICM) in some patients, illustrating the delicate balance between therapeutic benefit and unintended consequences [10].

VHD plays a crucial role in ventricular remodeling, hemodynamic deterioration, and worsening functional capacity. Transcatheter aortic valve implantation (TAVI) and transcatheter edge-to-edge repair (TEER) with MitraClip and TriClip have dramatically improved its management, significantly modifying ventricular loading conditions, myocardial metabolism, and structural remodeling [11].

This review aims to integrate current knowledge on the molecular adaptations induced by implantable cardiac technologies. It comprehensively examines all major devices within a disease- and intervention-specific framework, detailing the underlying mechanisms, geometric changes, and structural remodeling they induce. Rather than adopting a systematic review methodology, the manuscript provides a descriptive and integrative analysis of device-induced biological effects across different clinical scenarios and pathophysiological contexts. The goal is to highlight shared molecular pathways, identify condition-specific responses, and explore emerging strategies to enhance device performance while minimizing adverse effects on cardiac architecture. A deeper understanding of these processes is crucial for improving patient outcomes, refining implant selection criteria, and shaping the next generation of bioengineered cardiac solutions.

## 2. Ventricular Unloading

The decreasing ventricular filling and afterload is a key therapeutic goal that can be achieved in various clinical settings using both short- and long-term mechanical circulatory support devices, as well as transcatheter heart valve systems. The implantation of these technologies requires the collaboration of multiple specialized settings, including cardiac catheterization laboratories (Cath lab), cardiac surgery, and cardiac anesthesia. They are primarily used to manage critical conditions such as advanced HF, VHD, and cardiogenic shock, with the aim of optimizing hemodynamics and promoting myocardial recovery (Table 1).

### 2.1. Cath Lab

#### 2.1.1. Intra-Aortic Balloon Pump (IABP)

IABP reduces left ventricular (LV) afterload and enhances coronary perfusion. Through diastolic balloon inflation, it increases diastolic pressure, improving blood supply to the myocardium, while systolic deflation lowers aortic impedance, decreasing myocardial oxygen demand and end-systolic wall stress [12]. One of its key effects is the modulation of inflammation, as it has been associated with a reduction in pro-inflammatory cytokines such as tumor necrosis factor-alpha (TNF-α) and interleukin-6 (IL-6). Studies have demonstrated that patients receiving IABP therapy in combination with tirofiban during acute myocardial infarction experience a significant decrease in serum TNF-α and IL-6 levels, suggesting an anti-inflammatory effect that could help limit myocardial fibrosis and endothelial dysfunction [13]. Furthermore, IABP plays a role in improving endothelial function by promoting the production of nitric oxide (NO) through the activation of endothelial nitric oxide synthase (eNOS). NO is a crucial regulator of vascular tone and microcirculatory perfusion, and its increased availability during IABP support has been linked to improved organ function and reduced endothelial activation in the setting of pulsatile extracorporeal circulation [14]. In addition to its vascular and inflammatory benefits, IABP also influences coagulation and fibrinolysis, as it induces pulsatile blood flow, which has been shown to modulate coagulative and fibrinolytic responses during extracorporeal circulation, potentially reducing thrombotic complications [15]. Moreover, IABP therapy has been associated with an increase in coronary blood flow velocity in critically ill patients, enhancing oxygen delivery to the ischemic myocardium and supporting myocardial recovery [16]. Despite mixed clinical trial outcomes, IABP remains an essential tool for cardiogenic shock and high-risk percutaneous coronary intervention, with similar survival rates compared to percutaneous mechanical circulatory support [17]. Further studies are needed to optimize its use and identify biomarkers of response to unloading.

#### 2.1.2. Impella and Short-Term Mechanical Support

The Impella device provides temporary LV unloading, effectively reducing LV filling pressures and myocardial oxygen demand. At the molecular level, Impella-induced shear stress activates endothelial cells, leading to acquiring the von Willebrand disease due to the proteolysis of the von Willebrand factor by a disintegrin and metalloproteinase with a thrombospondin type 1 motif, member 13 [18]. While shear stress theoretically induced by Impella could activate nuclear factor kappa B (NF-κB), promoting endothelial dysfunction and inflammation, proteomic studies have demonstrated that Impella suppresses NF-κB, leading to a reduction in monocyte adhesion and migration through chemokine ligands 3 downregulation [19]. Further, recent studies indicate that Impella preserves diastolic coronary perfusion, supporting myocardial recovery rather than contributing to ischemic injury [20].

#### 2.1.3. Transcatheter Aortic Valve Implantation (TAVI)

AS imposes chronic pressure overload on the LV, leading to concentric hypertrophy, myocardial fibrosis, and diastolic dysfunction. Over time, excessive afterload also leads to LV stiffness, microvascular rarefaction, and contractile dysfunction, ultimately progressing to HF with preserved or reduced ejection fraction (EF). TAVI reduces afterload abruptly, triggering a series of biochemical, metabolic, and structural adaptations that define the remodeling process [21].

Myocardial alterations after TAVI occur in three key phases: acute, early, and long-term adaptation.

- Acute phase: within the first hours post-procedure, there is transient myocardial dysfunction affecting both systolic and diastolic components, as evidenced by changes in echocardiographic parameters (e.g., E/E′ ratio, left atrial volume, Myocardial performance index) and a rise in myocardial damage markers (Troponin I, CK-MB, ST2) [22]. Coronary circulation is also affected, with increased hyperemic flow velocity and modest reductions in microvascular resistance [23].
- Early remodeling (one month following TAVI): structural improvement of LV becomes evident, with reduced LV mass index and wall thickness, improved Doppler velocity indices, and increased aortic valve area [24]. Functional enhancement is also reflected in lower pulmonary systolic arterial pressure and an improved Kansas City Cardiomyopathy Questionnaire score, indicating better patient-reported outcomes [25].
- Long-term adaptation: further enhancements in myocardial function include improved flow-mediated dilation and further amelioration of diastolic and systolic function (e.g., LV mass index, LV EF, global longitudinal strain) [26,27]. Many patients experience New York Heart Association (NYHA) class improvement, though some remain unchanged [28].

At the structural level, the reversal of chronic pressure overload leads to gradual regression of LV hypertrophy. Studies show that LV mass decreases by 10–25% within the first year post-TAVI, primarily due to reduced interstitial fibrosis and myocardial water content, rather than direct cardiomyocyte atrophy [29]. The process is accompanied by improved myocardial energetics, as reflected by an increase in the phosphocreatine/adenosine triphosphate (ATP) ratio, indicative of restored mitochondrial function. At the molecular level, relief from afterload following TAVI leads to downregulation of hypertrophy-related pathways such as the calcineurin/Nuclear factor of activated T-cells (NFAT) axis and Mitogen-activated protein kinase signaling [30]. Additionally, Sarco-Endoplasmic Reticulum Calcium ATPase 2a (SERCA2a) expression and calcium handling improve, leading to better diastolic relaxation and contractile reserve. SERCA2a gene therapy demonstrated the ability to maintain sarcoplasmic reticulum calcium balance, lower cardiac ryanodine receptor type 2 (RyR2) phosphorylation and calcium leakage, and minimize cellular-triggered arrhythmic activity. In failing hearts, it has been shown to decrease both spontaneous and stress-induced VA [31].

However, upon TAVI, new-onset atrioventricular conduction delays often occur, and some conduction blocks, including high-degree cardiac arrhythmias, may be asymptomatic and paroxysmal [32]. Scar formation around the stent struts has been documented, and direct mechanical forces against aortic valve and surrounding tissues in the LV outflow tract might be associated with re-entrant foci for sustained tachyarrhythmias [33,34]. The fibrotic response to chronic pressure overload is a critical determinant of remodeling outcomes post-TAVI. Chronic AS is associated with excessive transforming growth factor β (TGFβ)-mediated fibrosis, leading to ECM expansion and ventricular stiffening [35]. After TAVI, a gradual rebalancing of matrix metalloproteinases (MMPs) and tissue inhibitor of metalloproteinase (TIMP) expression facilitates collagen turnover and fibrosis regression [36]. However, in patients with advanced myocardial fibrosis, the ability to remodel is often limited, leading to persistently impaired ventricular compliance and suboptimal functional recovery [37].

#### 2.1.4. Transcatheter Edge-to-Edge Repair with MitraClip

MR is a common consequence of ischemic and non-ischemic cardiomyopathy, where LV dilation, annular remodeling, and papillary muscle displacement contribute to malcoaptation of the mitral valve leaflets. The result is chronic volume overload, which promotes LV enlargement, eccentric hypertrophy, and increased wall stress, accelerating HF progression. TEER with MitraClip reduces MR and LV volume overload, initiating a process of reverse remodeling and functional recovery. In severe LV dilation and advanced MR, it may exhibit limited remodeling capacity, as chronic volume overload induces irreversible ECM expansion and papillary muscle dysfunction. Pre-procedural predictors of favorable reverse remodeling include smaller baseline LV dimensions, greater contractile reserve, and lower myocardial fibrosis burden. In patients with end-stage cardiomyopathy and severely reduced contractility, TEER may provide symptomatic benefit without significant ventricular recovery, reinforcing the importance of careful patient selection [38].

At the structural level, MitraClip leads to a significant reduction in MR, resulting in increased cardiac output and systolic pressure, while diastolic and pulmonary pressures remain stable. Echocardiographic assessments show decreased LV end-diastolic volume, a slight reduction in LVEF, and improved antegrade systolic output [39]. Most patients experience symptomatic relief, reflected in NYHA class reduction. Left atrial volume and strain improvements, though significant, are less pronounced than LV remodeling but are still associated with a reduction in arrhythmic burden [40,41]. The RV shows mild adaptation, with improved Tricuspid annular plane systolic excursion and tricuspid systolic velocity, but no significant dimensional changes [42]. Remodeling is more pronounced in patients with LVEF > 40%, while those with LVEF < 40% show less volume reduction but similar long-term outcomes [43].

At the molecular level, volume unloading after TEER is associated with the inhibition of stress-induced signaling pathways, which are linked to eccentric hypertrophy and chamber dilation. Among these, calcineurin/NFAT and CaMKII signaling—which drive cardiomyocyte hypertrophy, dilation, and collagen deposition—are downregulated. In this context, calcineurin inhibition prevents NFAT dephosphorylation and nuclear translocation, thereby suppressing fetal gene reactivation and pathological myocardial growth. Similarly, the downregulation of RyR2 activity in failing hearts reduces its hypersensitivity to calcium release, helping to mitigate delayed afterdepolarizations and VAs [44,45]. Further, changes in SERCA2a and RyR2 phosphorylation contribute to better intracellular calcium cycling, improving contractile efficiency and diastolic function [46].

The fibrotic response in functional MR differs from pressure-overload cardiomyopathy, as volume overload is associated with a more diffuse and interstitial fibrotic pattern. Following MitraClip implantation, reduced wall tension and improved myocardial perfusion promote gradual collagen remodeling, facilitated by rebalancing of MMP/TIMP activity [47]. From a geometric perspective, TEER procedure leads to significant alterations in mitral annular dimensions; however, these modifications are not directly associated with the immediate reduction in MR severity [48].

#### 2.1.5. Transcatheter Edge-to-Edge Repair with TriClip

TriClip therapy, a TEER system for tricuspid regurgitation, contributes to ventricular unloading by reducing right ventricular (RV) volume overload and improving LVEF through ventricular interdependence mechanisms [49]. By enhancing tricuspid valve competence, TriClip decreases RV dilation and wall stress, leading to reduced pulmonary venous congestion and indirectly improving LV filling and cardiac output. This reduction in RV preload and afterload alleviates interventricular septal shift, optimizing LV geometry and function [50].

Although direct molecular investigations specific to TriClip therapy are currently lacking, its physiological impact on RV remodeling offers a plausible mechanistic basis. By reducing tricuspid regurgitation (TR) and associated volume overload, TriClip attenuates wall stress and favorably influences pathways involved in oxidative stress, mitochondrial function, and ECM remodeling. These hypotheses are grounded in established pathophysiological models of right HF, and further studies may help elucidate the biological correlates of this therapeutic approach.

Chronic RV pressure and volume overload contribute to the overproduction of reactive oxygen species (ROS), which, in turn, promote mitochondrial dysfunction, endothelial damage, and cardiomyocyte apoptosis. Elevated ROS levels activate TGF-β signaling, stimulating fibroblast-to-myofibroblast differentiation and excessive collagen deposition, leading to ventricular stiffness and fibrosis. RV hypertrophy precedes fibrosis, with delayed collagen accumulation mediated by fibroblast activation. By reducing hemodynamic stress on the RV, TriClip may help mitigate ROS generation and TGF-β activity, thereby limiting ECM remodeling and preserving myocardial compliance [51,52].

Beyond oxidative stress, mitochondrial dysfunction plays a pivotal role in RV failure under conditions of prolonged pressure overload. Studies suggest that maintaining mitochondrial homeostasis, particularly through pathways regulating uncoupling protein 2, is essential for preserving cardiac energy metabolism and contractile efficiency. Inflammation is another key factor in RV remodeling, as chronic hemodynamic stress activates inflammatory cascades that accelerate myocyte apoptosis, fibrosis, and microvascular dysfunction. In experimental models, strategies for reducing RV overload may downregulate pro-inflammatory cytokines and fibrotic mediators, curbing the progression of adverse ventricular remodeling [53].

Neurohormonal activation, particularly via the renin–angiotensin–aldosterone system (RAAS) and natriuretic peptides, is another hallmark of RV dysfunction. The failing heart exhibits increased BNP and aldosterone levels, exacerbating fluid retention, ECM deposition, and adverse structural changes. By relieving systemic congestion, TriClip may attenuate RAAS activation, reducing aldosterone-mediated fibrosis and fostering a more favorable ventricular remodeling process [54].

### 2.2. Cardiac Surgery

#### Left Ventricular Assist Device (LVAD)

LVADs provide continuous mechanical unloading by actively pumping blood from the LV to the aorta, reducing LV end-diastolic pressure, volume, and myocardial oxygen consumption. This unloading leads to significant structural and molecular adaptations, including a shift in the pressure–volume loop from a rectangular to a triangular shape due to the loss of isovolumic contraction and relaxation phases. Higher LVAD speeds further decrease pulmonary capillary wedge pressure, enhance cardiac output, and may cause LV–aortic pressure uncoupling, leading to prolonged aortic valve closure. At the molecular level, LVAD supports pathological hypertrophy by downregulating hypertrophy-related genes, including GATA4 (GATA-binding protein 4), Myosin Heavy Chain 7, and Natriuretic Peptide A. It also improves calcium cycling by increasing SERCA2a expression, reducing RyR2 instability, and restoring Na^+^/Ca^2+^ exchangers 1 (NCX1) function. Mitochondrial remodeling is characterized by reduced mitochondrial DNA damage, enhanced oxidative phosphorylation, and restoration of ATP production. Despite these improvements, ECM remodeling is often incomplete, with increased collagen crosslinking due to dysregulated TIMP/MMP activity, contributing to persistent myocardial stiffness. Additionally, LVADs modulate immune responses by reducing myocardial IL-6 and TNF-α but maintaining systemic inflammation. While LVAD-induced reverse remodeling improves cardiac function, full myocardial recovery remains elusive, emphasizing the need for targeted therapies to enhance myocardial recovery [2,55].

### 2.3. Cardiac Anesthesia

#### Extracorporeal Membrane Oxygenation (ECMO)

ECMO supports systemic circulation in cardiogenic shock, but its hemodynamic effects on the myocardium are complex. ECMO increases LV afterload due to retrograde aortic flow, which can lead to LV distension, elevated myocardial wall stress, and pulmonary edema. This distension may hinder myocardial recovery and elevate the risk of intracardiac thrombosis [56]. Early recognition and management of LV distension are crucial to optimize patient outcomes. The interaction between blood components and the artificial surfaces of the ECMO circuit activates both inflammatory and coagulation cascades. This activation can result in systemic inflammatory response syndrome, characterized by elevated levels of cytokines such as IL-6, TNF-α, and interferon gamma. These inflammatory responses may lead to endothelial activation, increased vascular permeability, and microvascular dysfunction [57]. Systemic anticoagulation is essential during ECMO to prevent thrombotic complications within the circuit. Unfractionated heparin is commonly used; however, its application increases the risk of bleeding, including intracranial hemorrhage, surgical site bleeding, and gastrointestinal hemorrhage. Balancing anticoagulation to mitigate both bleeding and thrombotic risks remains a significant clinical challenge [18]. Advancements in ECMO technology have improved circuit biocompatibility, potentially reducing the activation of inflammatory and coagulation pathways; however, the need for systemic anticoagulation persists [58].

## 3. Ventricular Remodeling

Structural ventricular changes are a key process in HF, arrhythmias, and electrical dyssynchrony. CRT, CCM, and CSP strategies are utilized in the Electrophysiology laboratory to restore synchrony, enhance contractility, and mitigate adverse myocardial remodeling, improving clinical outcomes in affected patients (Table 2).

### 3.1. Electrophysiology Laboratory

#### 3.1.1. Conduction System Pacing (CSP)

PICM is a condition characterized by a decline in LVEF following chronic RV pacing, with prevalence estimates ranging from 6% to 22%. The pathophysiology of PICM is primarily driven by pacing-induced ventricular dyssynchrony, where bypassing the conduction system leads to slow, myocyte-to-myocyte propagation, interventricular and intraventricular dyssynchrony, and asymmetric workload distribution. This results in septal thinning, lateral wall hypertrophy, and impaired cardiac efficiency, ultimately contributing to progressive HF [59].

Altered myocardial metabolism plays a crucial role in PICM pathogenesis, leading to fibrosis, myofibrillar disarray, and ECM remodeling. While these changes are not detectable with standard imaging techniques, they can be assessed in tissue samples or circulation biomarkers. Experimental models show that RV apical pacing in dogs leads to lateral wall hypertrophy, ECM remodeling, and overexpression of collagen type II, along with increased levels of MMP-2, MMP-9, TIMP-1, and TIMP-3 [60]. Histological studies in both animal and human models confirm the presence of myofibrillar hypertrophy, fatty infiltration, and interstitial fibrosis, reinforcing the maladaptive structural consequences of prolonged RV pacing. Identifying at-risk patients using advanced imaging, such as global longitudinal strain, could allow for earlier intervention to prevent disease progression [61].

Leadless pacemakers, although eliminating lead-related complications, still follow a non-physiological activation sequence, leading to similar dyssynchronous effects. Physiological CSP, by contrast, preserve normal conduction, reducing fibrosis, dyssynchrony-induced hypertrophy, and LV dilation, resulting in improved contractility and a lower risk of HF worsening [62,63].

Beyond its physiological benefits, CSP via transvenous leads is associated with structural and thrombotic complications, including TR, venous thrombosis or occlusion, and, more rarely, cardiac perforation. Although traditionally regarded as mechanical sequelae, these adverse events increasingly appear to result from a complex interplay between anatomical disruption, endothelial injury, and local molecular responses induced by device implantation.

Lead-related TR results from interference with tricuspid leaflet coaptation, subvalvular entrapment, or progressive fibrotic remodeling at the valvular–lead interface. Recent echocardiographic evidence suggests that the severity of TR may significantly worsen following implantation of transvenous implantable cardioverter defibrillator (ICD) leads, with a similar trend observed in patients receiving RV-paced systems [64].

Venous thrombotic events, particularly involving the subclavian and brachiocephalic veins, have been documented in up to 30% of patients post-implantation. Prospective studies employing venography and biomarker analysis have demonstrated significant post-procedural elevations in prothrombin fragment 1 + 2, D-dimer, von Willebrand factor, and thrombomodulin, reflecting a transient prothrombotic and pro-inflammatory state. However, the onset of clinically significant venous lesions appears multifactorial, involving patient-specific thrombophilic predispositions and endothelial activation, rather than procedural variables alone [65,66].

Cardiac perforation remains an infrequent but potentially life-threatening complication. Its pathophysiology involves local myocardial vulnerability, anticoagulation status, and lead design. Early diagnosis and prompt intervention, typically percutaneous, are essential for favorable outcomes [67].

#### 3.1.2. Cardiac Resynchronization Therapy (CRT)

CRT improves ventricular synchrony in patients with HF and left bundle branch block, reducing myocardial stress and promoting structural, electrical, and functional reverse remodeling [68,69]. At the molecular level, CRT enhances calcium cycling by increasing SERCA2a activity and phospholamban phosphorylation, leading to improved intracellular calcium reuptake. Restoration of connexin-43 expression enhances electrical conduction, reducing re-entrant arrhythmias. CRT also downregulates TGF-β, decreasing fibrotic remodeling and ECM deposition [70].

At the structural level, CRT induces favorable changes in ventricular geometry, reducing LV end-diastolic volume and LV end-systolic volume. The improvement in contraction efficiency leads to a higher EF, contributing to symptomatic relief [71]. In non-responders, however, persistent fibrosis and myocardial stiffening may limit these effects [72], necessitating alternative pacing strategies, which provides a more physiological activation pattern and better preserves myocardial function [73].

CRT shares many of the lead-related complications observed by CSP. TR is predominantly attributed to mechanical interference with the tricuspid valve apparatus. However, comparative data indicate that biventricular pacing is associated with a lower incidence and progression of TR compared to RV pacing (9.8% vs. 17.4% for ≥II TR), with better preservation of RV systolic function. These results suggest that CRT attenuates lead-related TR progression, possibly through improved interventricular synchrony and reduced mechanical stress on the tricuspid annulus [64,74].

#### 3.1.3. Cardiac Contractility Modulation (CCM)

CCM is an emerging therapy for patients with LVEF with reduced or preserved EF [75,76]. Unlike pacing or resynchronization therapies, CCM delivers non-excitatory electrical impulses during the absolute refractory period, enhancing myocardial contractility without altering heart rhythm. This intervention exerts significant effects at both the molecular and structural levels, promoting adaptive remodeling and improving systolic function [77].

At the molecular level, preclinical models and in vitro experiments have demonstrated that CCM primarily enhances calcium cycling and contractile protein function. The stimulation increases phosphorylation of phospholamban (PLN), reducing its inhibitory effect on SERCA2a, leading to improved calcium reuptake into the sarcoplasmic reticulum and more efficient excitation–contraction coupling. Additionally, CCM upregulates NCX1, optimizing calcium homeostasis and improving cardiac contractility. CCM also influences gene transcription, promoting NFAT and GATA4 activation, which regulate the expression of sarcomeric proteins such as troponin I and myosin heavy chain. Unlike pacing, which can induce dysfunctional hypertrophy, CCM fosters a physiological hypertrophic response, improving myofibrillar organization and contractile efficiency [78,79]. These molecular mechanisms are consistent with the functional improvements documented in clinical settings. Nonetheless, their validation in human myocardial tissue is still limited, as much of the current understanding is derived from preclinical models or isolated cardiomyocyte studies. Additional translational research is needed to consolidate the mechanistic basis of CCM in human pathology.

The effects of CCM on ventricular structure are characterized by a reduction in LV chamber stiffness while preserving ventricular mass and wall thickness. As opposed to LVAD-induced unloading, which may lead to atrophy, CCM facilitates reverse remodeling by promoting increased myofibrillar density and improved myocardial compliance. In contrast to CRT, which primarily benefits patients with conduction delays, CCM provides contractile improvement even in patients without left bundle branch blocks, expanding its potential therapeutic role. Clinical trials have demonstrated reductions in LV end-diastolic pressure and pulmonary capillary wedge pressure, leading to symptomatic improvement and enhanced exercise capacity in HF patients [80,81].

Rather than altering cardiac geometry through direct resynchronization or unloading, CCM refines intracellular signaling pathways to enhance myocardial efficiency and mechanical performance. While it does not replace CRT or mechanical support in advanced HF, it represents a promising approach for patients with persistent symptoms despite optimal medical therapy. Ongoing research is exploring personalized signal modulation and hybrid therapies combining CCM with ICD or pharmacological interventions to maximize myocardial recovery [79,82].

## 4. Future Directions and Therapeutic Innovations

Despite the transformative impact of implantable cardiac devices on HF, arrhythmias, VHD, and ICM, their long-term interaction with the myocardium often triggers maladaptive remodeling that can limit therapeutic efficacy. As discussed in previous sections, common adverse adaptations include persistent fibrosis, which restricts myocardial recovery despite CRT or LVAD therapy; calcium handling abnormalities, contributing to PICM; chronic inflammation, exacerbated by mechanical circulatory support; and vascular remodeling, which affects coronary stents and endothelial function, increasing the risk of restenosis and thrombosis. Myocardial fibrosis arises from activated fibroblasts, inflammatory cytokines, and mechanical stress, leading to increased ventricular stiffness, impaired contractility, and electrical conduction abnormalities [83]. Given its pervasive impact on myocardial function, targeting fibrotic remodeling is essential for supporting decision-making or improving the long-term efficacy of implantable cardiac devices and advancing HF management [84] (Box 1).

Box 1 **Therapeutic implications based on molecular and structural effects of cardiac devices. List of abbreviations: CCM: cardiac contractility modulation, SERCA2a: Sarco/Endoplasmic Reticulum Calcium ATPase 2a, HF: heart failure, LVEF: left ventricular ejection fraction, CRT: cardiac resynchronization therapy, PLN: Phospholamban, TGF-β: transforming growth factor-β, HFrEF: heart failure with reduced ejection fraction, CSP: conduction system pacing, ECM: extracellular matrix, ECMO: Extracorporeal Membrane Oxygenation, LV: left ventricle, IABP: left ventricular assist device, LVAD: left ventricular assistant device, TAVI: transcatheter aortic valve implantation.**

CCM: Through non-excitatory electric stimulation, CCM upregulates SERCA2a and modulates calcium cycling proteins, improving systolic function. These molecular benefits underpin its indication in symptomatic HF patients with LVEF 25–45% who are not CRT candidates.

CRT: By restoring calcium homeostasis (via enhanced SERCA2a activity and PLN phosphorylation) and reducing fibrotic signaling (TGF-β downregulation), CRT reverses dyssynchrony-driven remodeling. These adaptations support its use in patients with HFrEF, wide QRS, and mechanical asynchrony to halt adverse geometric progression and improve contractile efficiency.

CSP: By preventing dyssynchronous activation, it preserves physiological fiber recruitment, mitigates maladaptive ECM remodeling, septal thinning, and lateral wall hypertrophy. Its structural preservation justifies preferential use in patients requiring high-burden ventricular pacing.

ECMO: Ensures systemic perfusion but exacerbates myocardial inflammation and LV overload in the absence of concomitant unloading. This informs its use in biventricular or refractory shock settings.

Impella/IABP: Prevents acute ischemic injury and reduces inflammatory cytokines. These effects support their early deployment in cardiogenic shock to preserve myocardial viability before irreversible damage ensues.

LVAD: Mechanical unloading reduces wall stress and normalizes metabolic gene expression, but prolonged use induces sarcomeric disassembly and mitochondrial dysfunction. Understanding these maladaptive patterns informs patient selection and adjunctive medical therapy during bridge-to-transplant or destination therapy.

MitraClip/TriClip: By reducing regurgitant volume and wall stress, these devices attenuate oxidative stress, ECM disruption, and right/left ventricular strain. Their benefit is maximal in patients with functional regurgitation and early-stage chamber dilation, where reverse remodeling is still achievable.

TAVI: Relief of pressure overload downregulates pro-fibrotic and pro-hypertrophic genes, favoring regression of concentric remodeling. These effects reinforce early intervention in severe aortic stenosis with incipient LV dysfunction.

The next generation of cardiac therapies aims to enhance myocardial recovery, minimize maladaptive remodeling, and improve device performance through gene therapy, AI-guided device optimization, telemedicine, bioactive implants, and advancements in transcatheter valve interventions [85,86,87]. Gene editing is being explored to modify fibrosis progression and improve myocardial compliance in patients with myocardial dysfunction. These technologies are being explored to downregulate pro-fibrotic signaling pathways, including TGF-β, with the goal of limiting ECM deposition and reducing myocardial stiffness [88]. Preclinical studies suggest that targeted genetic modulation of collagen synthesis could slow disease progression and fibrosis.

In further detail, AI, telemedicine, and machine learning are revolutionizing cardiac device management by enabling real-time arrhythmia risk prediction and automated pacing adjustments [89,90]. Specifically, AI-driven CRT programming can dynamically optimize atrioventricular intervals, improving synchronization and patient response rates [91]. Machine learning models trained on intracardiac electrograms and ECG data are being used to differentiate life-threatening arrhythmias from self-terminating rhythms, reducing unnecessary therapies [92]. The integration of wearable sensors and remote monitoring with AI-based analytics may further enhance HF management and improve survival [93,94].

Advancements in biomaterials and bioengineering are driving the development of bioactive cardiac devices designed to minimize inflammatory responses and improve long-term performance. Novel hydrogel-coated and bioresorbable polymer leads are being developed to reduce fibrotic encapsulation [95]. Nanoengineered stents are being developed to modulate endothelial function and prevent restenosis by promoting faster endothelial healing, reducing thrombosis, and limiting smooth muscle cells proliferation [96]. In mechanical circulatory support, pulsatile-flow LVADs are being developed to mimic physiological arterial pulsatility, improving vascular remodeling and end-organ perfusion compared to traditional continuous-flow LVADs [97].

Simultaneously, advancements in transcatheter valve interventions are improving durability, procedural precision, and long-term remodeling outcomes [98]. Regarding TAVI, next-generation polymer-based valve leaflets are being designed to increase durability and reduce structural valve degeneration, especially in younger patients [99,100]. Additionally, self-expanding and repositionable valve designs are being refined to improve coronary access and reduce paravalvular leaks [101]. Computational modeling is also being integrated to predict hemodynamic performance and optimize prosthesis selection based on patient-specific anatomy [102].

For TEER, ongoing advancements focus on improving leaflet capture mechanisms, reducing procedural complexity, and enhancing durability [103]. Novel chordal-sparing mitral valve replacement technologies aim to provide a more complete correction of MR while preserving native valve function, reducing long-term ventricular remodeling [104]. AI-assisted echocardiographic guidance is also being explored to enhance procedural precision, reducing complication rates and improving repair durability [105].

## 5. Conclusions

The implantation of cardiac devices significantly improves survival and quality of life in patients with HF, arrhythmias, and ICM, yet their long-term molecular and structural effects remain under investigation. While CRT and CSP promote reverse remodeling, ICD shocks, and chronic pacing, mechanical unloading can induce fibrosis, oxidative stress, and maladaptive myocardial changes. Stents and transcatheter valves restore hemodynamics but may trigger inflammatory and fibrotic responses, impacting long-term function.

Understanding the molecular mechanisms driving these adaptations is essential to predict and prevent adverse remodeling, improving device programming, patient selection, and long-term outcomes. Advances in gene therapy, AI-driven optimization, and bioengineered implants offer promising strategies to mitigate negative effects and enhance myocardial recovery. Future research must integrate molecular, biological, and engineering approaches to refine device therapies, ensuring maximal therapeutic benefit with minimal long-term myocardial stress.

## Figures and Tables

**Table 1:** Ventricular unloading: devices, mechanisms, and myocardial Effects.

Device/Procedure	Mechanism of Action	Molecular Effects	Structural/Functional Effects
IABP	↓ Afterload, ↑ coronary perfusion	↓ TNF-α/IL-6, ↑ NO (via eNOS), modulation of coagulation and fibrinolysis	↓ LV wall stress, ↑ coronary flow, improved endothelial function
Impella	Direct LV unloading via axial flow	↓ NF-κB, ↓ chemokine ligands, acquired vWF disease (via ADAMTS13 activation)	↓ LV pressure/volume, preserved perfusion, reduced oxygen demand
TAVI	Relief of pressure overload in aortic stenosis	↓ NFAT/calcineurin, ↓ TGF-β, ↑ SERCA2a, ↑ PCr/ATP ratio	↓ LV mass and fibrosis, improved diastolic/systolic function, reverse hypertrophy
MitraClip (TEER)	Reduction in MR and volume overload	↓ NFAT/CaMKII, ↓ RyR2 activity, ↑ SERCA2a, ↓ pro-arrhythmic signaling	↓ LVEDV, ↑ CO, improved NYHA class, partial reverse remodeling
TriClip (TEER)	Reduction in TR and RV volume overload	↓ ROS, ↓ TGF-β, ↓ RAAS, improved mitochondrial function	↓ RV dilation, improved interventricular interaction, indirect ↑ in LV filling and output
LVAD	Continuous mechanical LV unloading	↓ hypertrophic gene expression, ↑ SERCA2a, ↑ oxidative phosphorylation, ↓ IL-6/TNF-α	↓ LVEDP/volume, persistent ECM stiffness due to ↑ collagen crosslinking
ECMO	Systemic perfusion	↑ cytokines (IL-6, IFN-γ), systemic inflammation, endothelial activation	↑ LV wall stress if not vented, risk of pulmonary edema and thrombosis

List of abbreviations: IABP: intra-aortic balloon pump, TNF-α: tumor necrosis factor-alpha, IL-6: Interleukin 6, NO: nitric oxide, eNOS: Endothelial Nitric Oxide Synthase, LV: left ventricle, NF-κB: Nuclear factor kappa-light-chain-enhancer of activated B cells, vWF: Von Willebrand factor, ADAMTS13: a disintegrin-like metalloproteinase with thrombospondin motif type 1 member 13, NFAT: nuclear factor of activated T-cells, TGF-β: Transforming growth factor-β, CaMKII: Calcium/calmodulin-dependent protein kinase II, RyR2: Ryanodine Receptor 2, SERCA2a: sarco/endoplasmic reticulum Ca^2+^ adenosine triphosphatase-2a, PCr/ATP: phosphocreatine (PCr)/adenosine triphosphate ratio, TEER: Transcatheter Edge-to-Edge Repair, LVEDV: Left ventricular end-diastolic volume, CO: cardiac output, NYHA: New York Heart Association, ROS: reactive oxygen species, RAAS: renin–angiotensin–aldosterone system, RV: right ventricle, LVAD: left ventricular assist device, LVEDP: Left ventricular end-diastolic pressure, ECM: extracellular matrix, ECMO: extracorporeal membrane oxygenation, IFN-γ: interferon gamma, ↑ = increased; ↓ = decreased.

**Table 2:** Ventricular remodeling induced by cardiac device therapies.

Therapy/Device	Target Condition	Molecular Effects	Structural/Clinical Outcomes
CRT	HF with LBBB	↑ SERCA2a, ↑ PLN phosphorylation, ↑ Connexin-43, ↓ TGF-β	↓ LV volumes, ↑ EF, ↓ fibrosis, improved synchrony
Conduction System Pacing	PICM prevention, bradyarrhythmias	Physiological conduction system activation, ↓ MMP/TIMP imbalance, ↓ fibrotic signaling	↓ dyssynchrony, ↑ EF, better long-term remodeling
RV Apical Pacing	Bradycardia	↑ MMP-2/9, ↑ TIMP-1/3, ECM expansion, myofibrillar disarray	Septal thinning, lateral hypertrophy, ↓ contractility, ↑ fibrosis (PICM)
CCM	HF with reduced/preserved EF	↑ PLN phosphorylation, ↑ NCX1, ↑ GATA4/NFAT, ↑ myofibrillar organization	↓ chamber stiffness, preserved wall thickness, ↑ contractility, ↑ compliance

List of abbreviations: CRT: cardiac resynchronization therapy, HF: heart failure, LBBB: left bundle branch block, SERCA2a: sarco/endoplasmic reticulum Ca^2+^ adenosine triphosphatase-2a, PLN: Phospholamban, LV: left ventricle, EF: ejection fraction, TGF-β: Transforming growth factor-β, PICM: pacing-induced cardiomyopathy, MMP: metalloproteinases, TIMP: Tissue inhibitors of metalloproteinases, RV: right ventricle, ECM: extracellular matrix, CCM: cardiac contractility modulation, NCX1: sodium–calcium exchanger 1, GATA4: GATA-binding protein 4, NFAT: nuclear factor of activated T-cells, ↑ = increased; ↓ = decreased.

## Data Availability

No new data were created or analyzed in this study. Data sharing is not applicable.

## Abbreviations

The following abbreviations are used in this manuscript:

AF	Atrial Fibrillation
AI	Artificial Intelligence
ATP	Adenosine Triphosphate
BNP	B-type Natriuretic Peptide
CaMKII	Ca^2+^/Calmodulin-dependent Protein Kinase II
Cath lab	Catheterization Laboratory
CCM	Cardiac Contractility Modulation
CK-MB	Creatine Kinase-MB
CSP	Conduction System Pacing
CRT/CRT-D	Cardiac Resynchronization Therapy/with Defibrillator
ECM	Extracellular Matrix
ECMO	Extracorporeal Membrane Oxygenation
EF	Ejection Fraction
eNOS	Endothelial Nitric Oxide Synthase
GATA4	GATA-binding protein 4
HF	Heart Failure
ICD	Implantable Cardioverter Defibrillator
IL-1β/IL-6	Interleukin-1 beta/Interleukin-6
IABP	Intra-Aortic Balloon Pump
LV	Left Ventricle/Left Ventricular
LVAD	Left Ventricular Assist Device
LVEF	Left Ventricular Ejection Fraction
MMP/TIMP	Matrix Metalloproteinase/Tissue Inhibitor of Metalloproteinase
MR	Mitral Regurgitation
NCX1	Na^+^/Ca^2+^ Exchanger 1
NFAT	Nuclear Factor of Activated T-cells
NF-κB	Nuclear Factor kappa-light-chain-enhancer of activated B cells
NO	Nitric Oxide
NYHA	New York Heart Association
PICM	Pacing-Induced Cardiomyopathy
PLN	Phospholamban
RAAS	Renin–Angiotensin–Aldosterone System
ROS	Reactive Oxygen Species
RV	Right Ventricle/Right Ventricular
RyR2	Ryanodine Receptor type 2
SERCA2a	Sarco-Endoplasmic Reticulum Calcium ATPase 2a
ST2	Suppression of Tumorigenicity 2
TAVI	Transcatheter Aortic Valve Implantation
TEER	Transcatheter Edge-to-Edge Repair
TGF-β	Transforming Growth Factor-beta
TNF-α	Tumor Necrosis Factor-alpha
TR	Tricuspid Regurgitation
VA/VAs	Ventricular Arrhythmias
VHD	Valvular Heart Disease
